# The development and validation of an integrated assessment of irrational beliefs concerning basic psychological needs: the rational emotive self-determination scale for workers

**DOI:** 10.3389/fpsyt.2025.1571324

**Published:** 2025-06-06

**Authors:** Murat Artiran, Pinar Tinaz, Ömer Faruk Simsek, Anthony Miller, Katia Correa Vione, Martin James Turner

**Affiliations:** ^1^ Department of Psychology, Southern New Hampshire University, Manchester, NH, United States; ^2^ Department of Psychology, Beykoz University, Istanbul, Türkiye; ^3^ Department of Psychology, Istanbul Rumeli University, Istanbul, Türkiye; ^4^ School of Health, Education, Policing & Sciences, Staffordshire University, Stoke-on-Trent, United Kingdom; ^5^ Department of Psychology, University of Birmingham, Birmingham, United Kingdom; ^6^ School of Psychology, Manchester Metropolitan University, Manchester, United Kingdom

**Keywords:** self-determined motivation, irrational beliefs, psychometric development, basic psychological needs, occupational setting

## Abstract

**Introduction:**

Recently a new approach to understanding human behaviour has emerged that integrates rational emotive behaviour therapy (REBT) from the cognitive behavioural tradition, and self-determination theory (SDT) from the humanistic tradition. In the current study, we develop a psychometric that conceptualizes this new approach in organisational settings; the rational emotive self-determination scale for work (RESD-W). The RESD-W assesses respondents’ irrational beliefs (from REBT) concerning the basic psychological needs (from SDT), namely perceptions of autonomy, competence, and relatedness within their work life. The RESD-W builds on initial validations of the RESD in adolescent populations (RESD-A).

**Methods:**

In the current paper, the psychometric properties of the 16-item RESD-W were examined across five studies, in which the factor structure, the reliability of the scale, and construct and criterion-oriented validity of the RESD-W were assessed.

**Results:**

Analyses confirmed theoretical expectations and yielded good psychometric properties. Scores in the RESD-W were associated with anxiety and depression, and negative emotions in the workplace.

**Discussion:**

The results are discussed regarding practice, highlighting that work related psychological wellbeing may be predicated on the integration of irrational beliefs and basic psychological needs.

## Introduction

Cognitions, emotions, and behaviours that characterise human motivation have been of significant interest for many years (e.g., 1, 2). Occupational psychology is one of the primary areas in which concepts of motivation have been extensively investigated (e.g., 3). One such theory that investigates work-related motivation and behaviour is self-determination theory (SDT; 4). SDT (5–7) is a prominent metatheory of motivation that provides insights into human potential for achievement and wellbeing through a framework of six theories. Of the six mini-theories, the Basic Psychological Needs Theory (BPNT), Cognitive Evaluation Theory (CET) and Organismic Integration Theory (OIT) have received significant research attention (8). Taken together, BPNT, CET and OIT propose that psychological health can be determined by the quality of one’s motivation (9), and that the satisfaction of basic psychological needs (BPNs) is vital for the achievement and maintenance of good quality motivation, known as autonomous motivation (i.e., self-determined, 6).

Research into the BPNs indicates that increases in one’s BPN satisfaction can encourage autonomous motives in athletic (10), educational (11, 12) and workplace settings (13–16). The basic psychological needs include one’s perceptions of competence in fulfilling actions, autonomy over completing wanted actions, and a sense of relatedness to the environment and actions taken (9). In achieving such needs, motives for action are likely to be autonomous, improving satisfaction, happiness, and well-being at work (7, 17). Yet, there is little evidence to suggest that satisfaction of BPNs can improve quality of motivation (i.e., from controlled to autonomous forms of motivation), to in turn improve well-being and satisfaction at work (14, 18). Though sparse, research within therapeutic settings has intimated that rational emotive behavior therapy (REBT) may serve to positively influence self-determined motivation and BPNs through diminishing irrational beliefs, and fostering rational beliefs (e.g., 19).

REBT (20) is a cognitive-behavioral therapeutic approach that aims to challenge irrational beliefs (21) to ameliorate dysfunctional emotions (e.g., anxiety) and maladaptive behaviours (e.g., withdrawal). Within REBT, it is postulated that goal relevant and goal incongruent events (i.e., adversities) are not the direct cause of dysfunctional emotions and maladaptive behaviours, but rather, it is the beliefs one applies to the event that underpins these reactions (22). The beliefs individuals have, determine the adaptivity of their response to adversity, and REBT holds that these beliefs can be rational (flexible, logical, and non-extreme) and or irrational (rigid, illogical, and extreme) (23). In REBT, there are four core irrational beliefs that underpin mental illbeing, including; demandingness (e.g., “I must”), awfulizing (e.g., “It is terrible”), frustration intolerance (e.g., “I cannot stand it”), and self/other depreciation (e.g., “I am worthless/others will think I am worthless”). Rational beliefs are likely to associate with adaptive behaviours (approach focus; 24), which include; preference (e.g., “I want”), anti-awfulizing (e.g., “It is not terrible if I don’t”), frustration tolerance (e.g., “I can stand it”), and unconditional self-acceptance (e.g., “If I fail, it does not mean that I am worthless”). Hereto, it is unsurprising that irrational beliefs and motivation regulation are associated (i.e., 25, 26). One’s motivation to engage may be important in the development of irrational beliefs (i.e., “I feel guilty if I don’t work, therefore I must”, 6), and it may be so that the underlying reasons why the goal is being pursued, and one’s sense of autonomy over actions (i.e., BPNs), can either exacerbate, or reduce chances of irrational beliefs emerging and mental ill-health increasing (26). With existing concomitant rhetoric highlighting that irrational beliefs concerning ones BPNs are damaging for anti-social behaviour, anger and emotional distress (18), it is fruitful to understand the potential deleterious effects this concomitance may have on mental health of workers.

Humans spend much of their lives in the workplace, and yet in many cases, the workplace is where interpersonal conflict occurs and BPNs are thwarted (i.e., you’re told what to do; 27). As such, if these threats to our BPN’s are met with irrational beliefs that distort perceptions of the self, others, and the world, then emotional suffering will be exacerbated (e.g., 18). Specifically, it is not inadmissible for one to think that “it would be terrible” if their autonomy was restricted, or that they would “be completely worthless” if they were deemed incompetent at their job, or that a lack of connectedness with colleagues was “unbearable”. And so, this individual is likely to experience unhealthy and maladaptive emotions (e.g., 18). Here, one’s suffering is not only because their BPNs are not being satisfied, but also because these BPNs are aggrandised by irrational beliefs. In REBT, the situation (e.g., being restricted, being incompetent, being shunned) does not trigger unhealthy emotions alone, rather it is one’s irrational beliefs (e.g., awfulizing) about the situation that underpins unhealthy emotions (28, 29).

In sum, improving upon intrinsic motivation (e.g., personal development, healthy relationships, being part of community goals) in work settings may be a challenge when individuals present with irrational beliefs. Through combining REBT and SDT it may be possible to aid the development of BPNs through the disputation and promotion of rational beliefs (i.e., cognitive restructuring). However, the application of both SDT and REBT in the prevention of unhealthy emotions and behaviours is not possible without a theoretical model. In an attempt to generate this, Artiran et al. (18) integrated the four core irrational beliefs as proposed in REBT with the three basic psychological needs as proposed within SDT, forming a psychometric assessment of irrational beliefs about the three basic psychological needs. The rational emotive self-determination in adolescents (RESD-A) scale is a context-specific (based on three psychological needs) irrational beliefs scale about the three BPNs, for use with adolescents. The integration of REBT and SDT constructs is important in part because the BPNs have been shown to be important for mental health and psychological wellbeing (e.g., 30). Indeed, research has indicated that greater irrational beliefs concerning the BPNs were related to greater psychopathology in a cohort of adolescents (18, 31).

The RESD-A was validated for use with adolescents, and therefore should not be used with non-adolescent samples, because the context of the items is specific to the adolescent experience. As such, to aid the continued investigation of the theoretical integration of REBT and SDT, the RESD should be tested and validated across additional populations such as work samples. Indeed, recent recommendations (32) and research (33) express the need to consider the context in which the psychometric is developed, in the aim to provide an accurate understanding of the subpopulation. Furthermore, according to Ellis (22) situational or context-specific rational and irrational beliefs are stronger predictors of emotional disturbance than general or non-context-specific beliefs. Furthermore, the context in which the current work took place is important, because Turkey is host to large numbers of people suffering from mental health conditions (18% of the population; 34), but the research concerning the mental health of workers in Turkey is sparse. In the current paper we report the development and validity testing of the an occupational version (RESD-W) of the recently developed RESD-A scale, developed alongside and for Turkish citizens.

The RESD has three components that combine irrational beliefs and three BPNs: autonomy irrational beliefs (AIB), competence irrational beliefs (CIB), and relatedness irrational beliefs (RIB). As such, the RESD-A measures adolescents’ irrational beliefs concerning autonomy, competence, and relatedness with the four core irrational beliefs; demandingness, awfulizing, frustration intolerance, and depreciation. In the current research, the RESD-A is adapted for use in occupational settings, forming the RESD-Work (W) scale. In line with the development of the RESD-A, the present research tests the three-factor structure of the RESD-W. Specifically, the following is set out in 5 studies, representing five independent samples of participants. Scale development in the present studies align with psychometric questionnaire development guidelines for a latent variable approach (35):

Study 1, sample 1: Scale construction, item generation, conceptual consistency of the items, initial scale development.
Study 2, sample 2: Explanatory factor analysis and internal consistency of the RESD-W scale.
Study 3, sample 3: Confirmatory factor analysis of the RESD-W scale.
Study 4, sample 4: Testing construct and criterion-oriented validity of the RESD-W scale.
Study 5, sample 5: Test-retest reliability of the RESD-W scale.

## Study 1

### Method

For all studies in the current paper, data were collected in the Turkish cities of Istanbul and Ankara. Participants were selected via random (self-selecting) and convenience sampling, whereby the authors contacted organizations local to Istanbul and Ankara to offer them the opportunity to take part. There are a number of workplaces in Istanbul and Ankara, including banks, food distribution companies, and cafes and restaurants. Participation in the research was on a voluntary basis. All data were collected by interviewing the participants face-to-face, not online. The informed consent statement was read to the participants verbally, who were then given the opportunity to ask any questions about the study. This aim of the current study is to develop a new psychometric to assess irrational beliefs regarding the three basic psychological needs in occupational populations (RESD-W), by adapting the existing RESD-A. Ethical approval by the relevant university was gained prior to data collection, and all participants (*n* = 29) provided informed consent.

### Scale construction and item development

To aid the research team in developing conceptually consistent items for the RESD-W, we recruited 6 clinical psychologists, 3 occupational psychologists and 20 occupational workers (10 female and 10 male). Liaising with experts and workers, the items were scrutinized for their meanings, expressions, and theoretical assumptions. After being reviewed by three clinical psychologists and two occupational psychologists, the number of items was reduced from 32-items to 24-items. Another 3 clinical psychologists and an occupational psychologist and 20 workers rated the face validity of the scale and provided feedback on its design. They reviewed the clarity and appropriateness of each scale item. 4 out of 24 items were re-written during the process due to a lack of clarity. At this stage, the RESD-W consisted of 24-items that achieved face validity.

Scale items were developed using a deductive process. Here, we take the hypothetical meaning of a construct to guide for the formation of items (36). During this process we were mindful of the target population, and items were developed with those in mind (37). The RESD-W scale measures irrational beliefs regarding the need for autonomy, relatedness, and competence in work-life. In the course of the preparation of the scale, REBT and SDT theories were used to achieve this deductive goal. The BPNs (9, 38) and the four core irrational beliefs were considered for the development of items. Consistent with the RESD-A (18), a five-point Likert-type scale was chosen as the response format.

While the scale was being developed, four measurement tools have been examined closely; Irrational Beliefs Test (39, Turkish adaptation: 40), The Attitudes and Belief Scale-2 (41, 42), Turkish adaptation of Basic Psychological Needs Scale (6, 43) and Work-Related Irrational Beliefs (44). Initially, we developed 32-items (8 items for each construct) and then categorized items based on their similarity to construct definitions, before arriving at 24-items to go through to data collection and exploratory factor analysis (EFA).

## Study 2

Study 1 identified that the RESD-W scale was a legible, valid scale, that on the face of it, measured irrational beliefs concerning autonomy, competence and relatedness. As a result, Study 2 aimed to assess the three-factor structure of the RESD-W via exploratory factor analysis. Ethical approval by the relevant university was gained prior to data collection, and all participants provided informed consent.

### Method

#### Participants

One hundred and fifty-three (*n* = 153) participants completed the RESD-W, 102 females (66.7%), 49 males (32.0%) and 2 (1.3%) others. Participants were aged between 18–67 years (36.56+/-13.52), inclusive of those educated to primary and elementary level (*n* = 11, 7.2%), secondary school level (*n* = 6, 3.9%), bachelors degree level (*n* = 98, 64.1%) and masters degree level (*n* = 24, 15.7%). The sample includes non-employed (*n* = 44, 28.8%), part-time workers (*n* = 18, 11.8%), full-time workers (*n* = 77, 50.3%), temporary workers (*n* = 5, 3.3%), and project-based workers (*n* = 9, 3.4%). This includes individuals working in limited companies (*n* = 71, 46.7%), family corporations (*n* = 14, 9.2%), as sole proprietors (*n* = 16, 10.5%) and freelancers (*n* = 8, 5.3%). These individuals worked either indoors (*n* = 94, 63.1%), outdoors (*n* = 31, 23.8%), or both indoors and outdoors (*n* = 22, 5.9%).

#### Adequacy of sample

The adequacy of the sample can be tested via the Kaiser-Meyer-Olkin (KMO; 45) statistic, with figures over.50 being considered suitable for factor analysis (46). In addition, the Bartlett’s test of Sphericity statistic must be significant (*p* <.05) for factor analysis to be possible with the given sample (47). The results of the KMO (Kaiser-Meyer-Olkin) coefficient (.769) and the Bartlett Sphericity Test (*p* <.05) determined that the sample size was suitable for analyses. To evaluate the factor structure of the RESD-W Scale, a principal axis factor analysis was executed on the 24 items. The number of factors to be extracted was then determined by (a) eigenvalues above 1, (b) Cattell’s scree-test, and (c) parallel analysis (48). Parallel analysis is a method applied to decide the number of factors by comparing the size of the eigenvalues with those produced by a randomly achieved data set.

#### Measures

The RESD-W comprised the 24-items developed in study 1, each of which are scored on a 5-point Likert-scale (1= not agree, 2= somewhat not agree, 3= somewhat agree, 4= agree, 5= definitely agree).

#### Analysis

Exploratory factor analysis (EFA) was conducted using Lisrel 8.51 was used to explore the underlying factor structure of the RESD-W. Namely, the adequacy of items for AIB, CIB, and RIB were assessed. In addition, the internal consistency of AIB, CIB and RIB was assessed using Cronbach’s *α* and McDonald’s ω. A *p*-value of < .05 with an α-level of 5% was set for statistical significance. The principal axis factoring method was conducted using the rotation technique of direct oblimin.

### Results

#### Exploratory factor analysis

For the 24 items, three factors greater than the value of 1 emerged, explaining 53.13% of total variance. As such, the RESD-W scale items were gathered in a three-factor structure. Results identify that the three-factor structure strongly fit the theoretical expectation of basic psychological needs related irrational beliefs when 16 items were included, down from 24 items. Factor loadings from the 16 remaining items can be seen in **Table 1**. Internal consistency (α, ω) (95% CI) of the RESD-W subscales were: AIB α = .81 (.76,.85), ω = .82 (.76,.86); CIB α = .72 (.64,.80), ω = .74 (.67,.81); RIB α = .65 (.57,.72), ω = .68 (.61,.74); Total α = .80 (.75,.84), ω = .80 (.69,.84).

**Table 1:** Exploratory factor analysis results: items and factor loadings.

Items	Factor 1	Factor 2	Factor 3
RSD7	.871		
RSD2	.789		
RSD1	.759		
RSD8	.492		
RSD5	.462		
RSD11	.399		
RSD12		.864	
RSD10		.731	
RSD9		.492	
RSD13		.487	
RSD16		.413	
RSD15			-.789
RSD14			-.747
RSD4			-.547
RSD6			-.449
RSD3			-.400

Factor 1: Irrational beliefs on competence needs (AIB’s), Factor 2: Irrational beliefs on autonomy needs (CIB’s), Factor 3: Irrational beliefs on relatedness needs (RIB’s).

## Study 3

Study 2 corroborated the findings of study 1, providing statistical support for the three-factor structure of the RESD-W. In study 3, we utilise a separate sample of participants in the attempt to confirm the three-factor structure of the RESD-W. Ethical approval by the relevant university was gained prior to data collection, and all participants provided informed consent.

### Method

#### Participants

One hundred and sixty-two (*n* = 162) participants completed the RESD-W, 77 females (47.5%), 70 males (43.2%) and 15 (9.3%) undisclosed. 10 participants for each variable in the model (49–52) is recommended, thus the sample size was acceptable for analyses. Participants were aged 18–44 years (30.07+/-5.60), inclusive of those who have no formal education (*n* = 14, 8.6%), primary and elementary education (*n* = 35, 21.6%), secondary education (*n* = 30, 18.5%), bachelors degree education (*n* = 67, 41.4%), and masters degree education (*n* = 16, 9.9%). The sample includes non-employed (*n* = 17, 10.5%), part-time workers (*n* = 24, 14.8%), full-time workers (*n* = 104, 64.6%), temporary workers (*n* = 8, 4.9%), and project-based workers (*n* = 9, 5.6%), being either indoor workers (*n* = 105, 64.8%), outdoor workers (*n* = 28, 17.3%), or both indoor and outdoor workers (*n* = 29, 17.9%).

#### Analysis

In order to confirm the factor structure of the RESD-W, confirmatory factor analyses (CFA) was completed using the maximum likelihood estimation method with Lisrel 8.51 (53). Several alternative indexes of fit as adjuncts to the Chi-square statistic were used (54), including the Chi-square to degrees of freedom ratio (x2/df), the comparative fit index (CFI), goodness-of-fit index (GFI), standardized root-mean-square residual (SRMR) and root-mean-square error of approximation (RMSEA). In this study, the most used indices of goodness of fit statistics considered were Incremental Fit Index (IFI), CFI, RMSEA, and SRMR. The values of.90 or greater for the indices indicate good model fit, and for SRMR and RMSEA, values of.08 or less indicate good fit (55–57). Additionally, we inspected the average shared variance (AVE) and shared variances (AVE-SV) of latent variables, respectively, based on Fornell and Larcker’s (58) recommendations to assess convergent and discriminant validity. There is sufficient evidence of convergent validity when the latent variable shares at least half of the variance with its own indicators, that is, the AVE should be at least.50. Discriminant validity is evidenced when the AVE is greater than the shared variance between latent variables.

### Results

**Figure 1:**
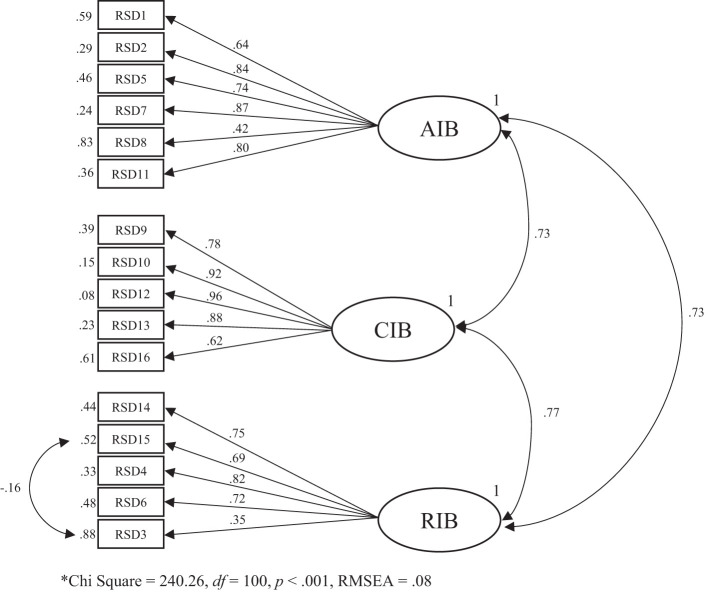
CFA standardized solution*.

**Figure 2:**
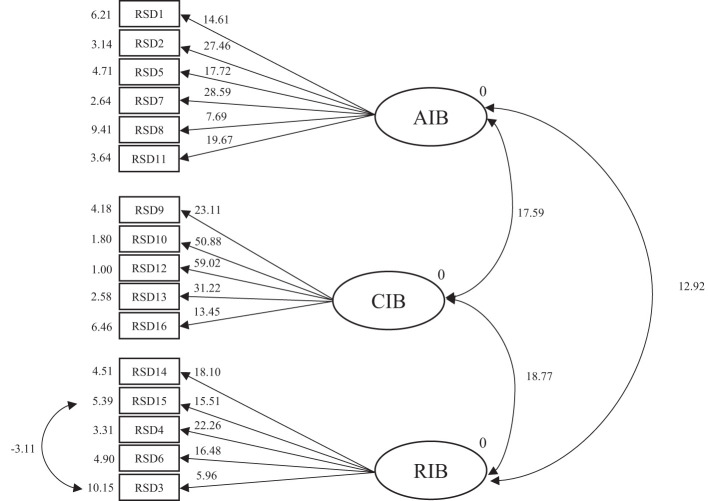
CFA T-values.

CFA analysis revealed that Goodness of Fit Index (.95), Comparative Fit Index (.94) Incremental Fit Index (.94), Root Mean Square Error of Approximation (.08), and Chi-square (203.11) were at least acceptable (**Figure 1**). Intercorrelations (covariances) between the three factors were moderate to high; RIB and AIB (*r* = .73, *p* <.05), RIB and CIB (*r* = .77, *p* <.05), and AIB and CIB (*r* = .73, *p* <.05). Examination of the standardized solution outcomes revealed no problems in the model coefficients. In the model, *t*-test values range from 5.96 to 59.02 and were statistically significant (**Figure 2**). Internal consistency (α, ω) (95% CI) of the RESD-W subscales were: AIB α = .75 (.69,.82), ω = .77 (.71,.82); CIB α = .83 (.79,.87), ω = .85 (.81,.89); RIB α = .70 (.63,.77), ω = .72 (.65,.78); Total α = .84 (.79,.88), ω = .82 (.78, 86). Convergent validity, based on the AVE was satisfactory for AIB (.54) and CIB (.59), but not for RIB (.39). Regarding discriminant validity, the shared variance (SV) between all pairs was lower than the AVE, indicating discriminant validity: AIB-CIB (SV = .11), AIB-RIB (SV = .09), and CIB-RIB (SV = .21).

## Study 4

Study 3 corroborated the findings of study 2, confirming the three-factor structure of the RESD-W. In study 4, we utilise another separate sample of participants in the attempt to test the construct and criterion-oriented validity of the RESD-W. We approach construct validity in two ways. First, to assess convergent validity we examine the bivariate relationships between RESD-W scores and basic psychological needs satisfaction to assess convergent validity. We hypothesise that positive associations will occur between subscales of the RESD-W and their counterpart basic psychological needs satisfaction. In other words, for example CIB should positively relate to competence need satisfaction because of their thematic overlap. Second, to assess discriminant validity we examine the bivariate relationships between RESD-W scores and positive work emotions, with the hypothesis that subscale scores on the RESD-W will not be related to positive work emotions. It is not anticipated that irrational beliefs would relate to positive emotions, because in the theory of REBT irrational beliefs are considered to underpin greater dysfunctional negative emotion and lower functional negative emotion (i.e., binary theory of distress; 59), rather than less positive emotion. That is, irrational beliefs are thought to relate to negative outcomes, whilst rational beliefs are thought to relate to positive outcomes (60). In addition, research has shown no association between irrational beliefs and positive affect (see 61). For criterion-oreinted validity we examine the bivariate relationships between RESD-W scores and psychological distress, depression, negative work emotions, and need for absolute truth to assess concurrent validity. We hypothesise that positive associations will occur between subscales of the RESD-W and psychological distress, depression, negative work emotions, and need for absolute truth.

### Method

#### Participants

One hundred and sixty-eight (*n* = 168) participants completed the questionnaires, 86 females (51.2%), 66 males (39.3%), 16 undisclosed (9.5%). Participants were aged 18–39 years (29.62 +/- 5.36), inclusive of those who have no formal education (*n* = 13, 7.7%), primary and elementary education (*n* = 26, 15.4%), secondary education (*n* = 33, 19.6%), bachelors degree education (*n* = 79, 47%), and masters degree education (*n* = 17, 10.1%). The sample includes non-employed (*n* = 13, 7.7%), part-time workers (*n* = 21, 12.5%), full-time workers (*n* = 121, 72%), temporary workers (*n* = 4, 2.4%), and project-based workers (*n* = 9, 5.4%). This includes individuals working in limited companies (*n* = 71, 46.7%), family corporations (*n* = 14, 9.2%), as sole proprietors (*n* = 16, 10.5%) and freelancers (*n* = 8, 5.3%). These individuals worked either indoors (*n* = 123, 73.2%), outdoors (*n* = 21, 12.5%), or both indoor and outdoor (*n* = 24, 14.3%). Ethical approval by the relevant university was gained prior to data collection, and all participants provided informed consent.

#### Analysis

Construct validity was investigated by calculating the Pearson’s bivariate correlation coefficients (*r*) (with sex partialled out) between RESD-W subscales and basic psychological need satisfaction (convergent) and positive work emotion (discriminant). Criterion-oriented validity was investigated by calculating the Pearson’s bivariate correlation coefficients (*r*) (with sex partialled out) between RESD-W subscales and psychological distress, depression, negative work emotions, need for absolute truth (concurrent; see 62). To interpret its *r* values, scores of.70 are considered a strong relationship,.50 as a moderate relationship, and.30 as a weak relationship (63).

#### Measures

##### Rational emotive self determination scale-work

The RESD-W consists of 3 dimensions; autonomy irrational beliefs (AIB), competence irrational beliefs (CIB), relatedness irrational beliefs (RIB). The scale consists of 16-items which measure irrational beliefs concerning the three basic psychological needs of workers. The scale uses a 5-point Likert scale (1= not agree, 2= somewhat not agree, 3= somewhat agree, 4= agree, 5= definitely agree). Internal consistency (α, ω) (95% CI) of the RESD-W subscales were: AIB α = .70 (.62,.78), ω = .72 (.64,.80); CIB α = .67 (.56,.75), ω = .68 (.58,.76); RIB α = .71 (.62,.77), ω = .73 (.66,.78); Total α = .83 (.79,.88), ω = .84 (.79, 88). The 16 items can be seen below in **Table 2**.

**Table 2:** Items within the RESD-W.

1	In work, I must be in control and willing when doing tasks, and nobody should interfere with me
2	If I can not work towards my own values and interests, it will be awful and I think it is like the end of the world
3	Even if I can’t get along with the others in my job I still believe they are valuable human beings
4	If I can not establish sincere and close relationships in my job, I can not stand it, it is unbearable
5	If people interfere with each other in a workplace, I believe completing the tasks have no worth
6	Without good and sincere human relationships in the workplace, it isn’t worth completing the tasks
7	I can’t stand it if I am not allowed to make my own decisions when I am doing my job, I believe it is unbearable
8	It is okay not working independently and willingly
9	If one can not do a task well enough, he/she should definitely stop doing it
10	It is awful if I am incompetent in my work life; I start thinking as if it is the end of the world
11	People who interfere in my will and my own decisions while I am doing my job are worthless in my eyes
12	I can not tolerate being insufficient and incompetent, I think such feelings are unbearable
13	The value I give to myself as an individual disappears when I am inadequate in my job
14	It is a ‘must’ that relationships with others in work place/life are sincere and warm
15	Whenever I feel distance and coldness from others in work life, I believe it is awful and I start thinking as if it is the end of the world
16	Being incompetent and inadequate in my job can be a bad thing, but it is not an awful thing

Items 3, 8, and 16 are reverse-scored.

##### The basic psychological needs support

The BPNS addresses need satisfaction in ones relationships (e.g., spouse, best friend, mother; 64). The BPNS has 9-items assessing the three needs: competence, autonomy, and relatedness (65). Because data collection was in Turkey, we found a suitable 9-item scale measuring psychological needs, that was then contextualised to the workplace, being valid and reliable in Turkish (43, 65). Here, items were contextualised from friendship groups to the workplace (e.g., “*When I am with my friends, I feel free to act as I am*” to “*When I am with my colleagues, I feel free to act as I am*”). Items were scored on a 5-point Likert-scale between 1 (not at all true) and 5 (very true). In the present study, following translation to Turkish (replicating 43), the Cronbach’s *α* reliabilities were; autonomy *α* = .66, competence *α* = .63, and relatedness *α* = .63.

##### Brief symptom inventory

The 90-item BSI (66) consists of nine subscales (somatization, obsessive-compulsive disorder, interpersonal sensitivity, depression, anxiety, hostility, phobic anxiety, paranoid ideations, psychoticism) and was translated into Turkish (67). Responses are assessed on a 5-point Likert-type scale from 0 (not at all) to 4 (extremely). Only total scores for anxiety and depression were used. The reliability coefficients of the subscales were between.71 and.85.

##### Needs for absolute truth

NAT measures the need to find absolute truth about oneself (68). NAT consists of 5-items. Responses are assessed on a 5-point Likert-type scale from 1 (not at all true) to 5 (very true). Cronbach Alpha was *α* = .74.

##### Job-related affective well-being scale

The Turkish-translated JAWS (69, 70) was used. The scale consists of 30-items, rating how often they feel specific emotions in their work-life from 1 (*Never*) to 5 (*Always*). Positive and negative emotion scores were evaluated separately. The sub-scales include: High pleasure-High arousal (HPHA; feelings of being energetic, excited, ecstatic, enthusiastic, inspired), High pleasure-Low arousal (HPLA; at-ease, calm, content, satisfied, relaxed), Low pleasure-High arousal (LPHA; anger, anxious, disgust, fright, furious), and Low pleasure-Low arousal (LPLA; bored, depressed, discouraged, gloomy and fatigued). Items are assessed on a 5-point Likert-scale from 1 (never) to 5 (extremely often), and scores for positive emotions are summed to form a positive work subscale, and scored for negative emotions are summed to form a negative work emotion subscale. Internal consistency coefficients of the scale were.92 for positive emotions and.93 for negative emotions.

##### Beck depression ınventory

The Turkish Translated (71, 72) Beck Depression Inventory (BDI) was used. Hisli (72) has determined reliability of the measure in Turkish. Each of the 20-items were assessed on a 4-point Likert-scale from 0 to 3 with scored summed to create a total depression score.

### Results

In order to determine construct and criterion-oriented validity of the RESD-W, we tested the association between the RESD-W and basic psychological needs satisfaction (BPNS), anxiety, need for absolute truth (NAT), psychological distress (anxiety and depression; BSI), depression (BDI), and positive and negative emotions in work-life (JAWS). See **Table 3** for intercorrelation matrix. For convergent validity, as hypothesised CIB was positively and strongly associated with competence need satisfaction (*r* = .80, *p* <.001), AIB was positively and strongly associated with autonomy need satisfaction (*r* = .88, *p* <.001), and RIB was positively and strongly associated with relatedness need satisfaction (*r* = .82, *p* <.001). For discriminant validity, only CIB was related to positive work emotion, and this associated was the weakest significant correlation in the analyses (*r* = -.18).

**Table 3:** Correlations between RESD-W and psychological variables.

variable	M	SD	1	2	3	4	5	6	7	8	9	10	11	12	13	14
1. AIB	18.76	4.60	1													
2. CIB	15.48	3.66	.39^**^	1												
3. RIB	15.01	3.92	.52^**^	.53^**^	1											
4. RSTOT	49.25	9.77	.80^**^	.78^**^	.84^**^	1										
5. ANX	25.55	10.00	.40^**^	.26^**^	.32^**^	.40^**^	1									
6. DEP	26.49	10.66	.42^**^	.26^**^	.32^**^	.42^**^	.85^**^	1								
7. NATTOT	14.98	5.33	.22^*^	.20^*^	.20^*^	.26^**^	.17^^^	.23^**^	1							
8. BSNAUT	11.58	2.72	.88^**^	.35^**^	.47^**^	.71^**^	.34^**^	.38^**^	.27^*^	1						
9. BSNCOM	11.67	2.49	.35^**^	.80^**^	.54^**^	.68^**^	.22^*^	.21^*^	.18^	.35^**^	1					
10. BSNRLT	10.99	2.64	.45^**^	.49^**^	.81^**^	.72^**^	.20^*^	.15^	.13	.43^**^	.57^**^	1				
11. BSNTOT	34.25	7.18	.73^**^	.67^**^	.76^**^	.89^**^	.33^**^	.32^**^	.25^**^	.78^**^	.77^**^	.83^**^	1			
12. NEG	36.00	12.34	.24^**^	.29^**^	.27^**^	.33^**^	.52^**^	.46^**^	.05	.25^*^	.21^*^	.27^**^	.31^**^	1		
13. POS	47.92	12.16	.03	-.18^	-.02	-.07	-.23^*^	-.28^**^	-.07	-.07	-.16^	-.11	-.14	-.47^**^	1	
14. BDI	35.83	13.91	.41^**^	.28^**^	.32^**^	.42^**^	.94^**^	.97^**^	.21^*^	.37^**^	.24^*^	.17^	.33^**^	.50^**^	-.28^**^	1

*n* = 168. ^ p <.05 * *p* <.01 ** *p* <.001. AIB, autonomy irrational beliefs; CIB, competence irrational beliefs; RIB, relatedness irrational beliefs; RSTOT, total score of RESD-W scale; ANX, brief symptom inventory anxiety scores on the BSI; DEP, brief symptom inventory depression scores on the BSI; NATTOT, total score of need for absolute truth scale; BSNAUT, basic psychological needs support of autonomy; BSNCOM, basic psychological needs support of competence; BSNRLT, basic psychological needs support of relatedness; BSNTOT, total score of basic psychological needs support scale; NEG, total score of negative emotions on JAWS; POS, total score of positive emotions on JAWS; BDI, Beck depression inventory scores.

For concurrent validity, RESD-W subscales were positively and weakly-moderately associated with psychological distress (anxiety: *r* = .26-.40; depression: *r* = .26-.42), negative work emotion (*r* = .24-.29), and depression as measured via the BDI (*r* = .28-.41), and positively and weakly associated with need for absolute truth (*r* = .21-.23).

### Discussion

Study 4 data analyses revealed findings that were largely consistent with hypotheses, but also some counter-indications of construct validity. RESD-W subscales were related to their basic psychological need satisfaction counterparts as hypothesised. Aside from convergent validity, the relationships between RESD-W subscales and basic psychological need satisfaction might also tell us about the construct itself. That is, the finding that each RESD-W subscale strongly related to its need satisfaction counterpart might indicate that irrational beliefs about the basic needs do not necessarily preclude workers attained the satisfaction of those needs. This is important because it might be possible to satisfy one’s basic psychological needs, but still experience psychological suffering (i.e., depression) due to the proposed effects of irrational beliefs about those needs. An addition, the strong associations between RESD-W subscales and the basic needs found in the present study might be driven by the questions on the RESD-W and BPNS questionnaires being similar in themes. That is, because the CIB questions of the RESD-W concern competence, as do the competence subscale questions in the BPNS, respondents might be more likely to score them in a similar manner. For discriminant validity, there was some support for the hypothesis that RESD-W subscales should be unrelated to positive work emotion, but CIB was very weakly related.

For criterion-oriented (concurrent) validity, RESD-W subscales were again related to outcome variables as hypothesised, but not strongly. However, it might be the case that irrational beliefs alone are not always strongly related to mental health outcomes, and indeed in the wider literature concerning the associations between irrational beliefs and mental health, a range of mediators have been hypothesized and evidenced. For example, Mansell and Turner (73) reported that irrational beliefs are related to psychological distress through self-confidence, whilst alternative mechanisms have been proposed including self-determined motivation (26), maladaptive schema (74) and negative automatic thoughts (75). Another proposed mechanism for the relationship between irrational beliefs and psychological distress are challenge and threat evaluations (61, 76, 77). In addition, whilst concurrent validity tests do indicate relationships between RESD-W subscales and important psychological outcomes, we cannot argue causal relationships or predictive validity, because we do not have temporal data in the study. Thus, although the results of study 4 are in the direction expected, they only provide tentative evidence of construct (convergent and discriminant) and criterion-orientedooriented (concurrent) validity.

## Study 5

Study 4 offered some evidence for the criterion-oriented validity of the RESD-W. In study 5, we utilise another separate sample of participants to examine the test-retest reliability of the RESD-W. Test-retest reliability is important as it indicates the reproducibility of a measure (78). We hypothesised that RESD-W subscale scores would be stable between two timepoints, two weeks apart. Ethical approval was gained by the relevant university prior to data collection, and all participants provided informed consent.

### Method

#### Participants

At least 50 participants at two time-points are recommended to detect an estimated intra-class coefficients (ICC) of.80 with a 95% confidence interval (CI) from.70 to.90 (79). Law (78) suggests ICCs of at least 0.75 with lower limits of CIs above.60, and an overall sample size of at least 30 participants. Thus, informed also by sampling guidelines form Polit (80), we recruited 56 participants to complete the RESD-W at two separate timepoints, two weeks apart. Of the 56 participants, 27 were female (47.7%), and 29 were male (52.3%). Participants were aged 21 to 33 years (25.00 +/- 3.66). The sample included non-employed (*n* = 5, 8.9%), part-time workers (*n* = 8, 14.3%), full-time workers (*n* = 41, 73.2%), temporary workers (*n* = 2, 3.6%). The education level of the participants was as follows: masters degree (*n* = 23, 41.1%), bachelors’ degree (*n* = 15, 26.8%), secondary education (*n* = 9, 16.1%), elementary and primary education (*n* = 9, 16.1%).

#### Procedures

All participants took part on a voluntary basis. The RESD-W was completed two-week apart from one another, following typical convention (52, 80, 81). Test–retest reliability was conducted using Pearson product–moment correlation coefficients and intra-class coefficients (ICC) to examine the associations among the subscales at the two timepoints. ICC and CI ranges between 0.5 and 0.75 indicate moderate reliability, values between 0.75 and 0.9 indicate good reliability, and values greater than 0.90 indicate excellent reliability (82) whilst a Pearson’s correlation coefficient of.70 indicates test–retest reliability (83).

### Results

The results revealed moderate-good test–retest reliability indicated by large, significant, positive correlation coefficients (RESD-W Total, *r* = .84, *p* <.01; AIB, *r* = .82, *p* <.01; CIB, *r* = .78, *p* <.01; and RIB, *r* = .80, *p* <.01), and sufficient ICC value. For AIB we found an ICC of.82 (CI,.72-.89), for CIB we found an ICC of.79 (CI,.66-.90), for RIB we found an ICC of.80 (CI,.69-.88), and for RESA-W total we found an ICC of.84 (CI,.75-.91).

## General discussion

The aim of the present paper was to develop and test the validity and reliability of the RESD-W. This five-study paper included; scale construction and item generation, testing of conceptual consistency of the items, exploratory factor analysis, checks for internal consistency, confirmatory factor analysis, tests for construct and criterion-oriented validity, and test re-test reliability, within five separate samples.

EFA and CFA results confirmed the three-factor structure of the RESD-W; AIB, CIB, and RIB. The scale measures workers’ irrational beliefs about three psychological needs (autonomy, competence, and relatedness) with a set of irrational belief items of demandingness, awfulizing, frustration intolerance and self-others-life (work, colleagues, or tasks) depreciation. Ryan and Deci (30) assert that human motivation and happiness depends on the three basic psychological needs autonomy, competence, relatedness (30). As such, it can be suggested that the RESD-W scale is coherent with SDT’s theoretical bases (i.e., a three-factor structure). The results of the current study are consistent with the extant research (18, 31), confirming the three distinct structures; autonomy irrational beliefs, competence irrational beliefs and relatedness irrational beliefs. Additionally, CFA results also provided support of convergent validity for AIB and CIB subscales and less favourable for RIB. The low AVE for RIB could be a result AVE’s tendency to penalise factor loadings below.80 (84). There was also initial support of discriminant validity between all latent variables (sub-scales). Based on these results and limitations of assessing convergent and discriminant validity based on AVE, further evidence of convergent and discriminant validity based on external latent variables was sought in Study 4. The reliability coefficients provided acceptable internal consistencies (85) for the dimensions and the whole scale of the RESD-W. Test-retest analysis indicated that the test–retest reliability was acceptable.

The three-factor structure of the RESD-W scale replicates that of the RESD-A (Rational Emotive Self Determination Scale for Adolescents): AIB, CIB and RIB (18). As such, the present research affirms the consistency of the three types of irrational beliefs, and results also indicate that a total RESD-W score is valid and useful in its relationship with important mental health outcomes. Indeed, whilst each subscale of the RESD-W offers some important insight into the nature of an individual’s irrational beliefs, the total score provides an overall indication of the extent to which a person applies irrational beliefs to the three basic psychological needs. Like in other irrational beliefs measures (e.g., irrational performance beliefs inventory; 86), this total score offers brevity in reporting, but using each subscale score is likely to be more useful for granular detail in research, and needs analysis in practice. That is, by using the three separate subscale scores it is possible for one to identify which basic psychological need a respondent is applying irrational beliefs to, thus helping the practitioner to structure their intervention work.

Of interest though, the RESD-W was positively associated with finding the absolute truth about the self, anxiety and depression, and negative emotions in the workplace. This result is perhaps unsurprising. If a worker holds strong rigid and unrealistic (irrational) beliefs regarding their ability to work (competence), freedom to work (autonomy), and feelings about the people I work with (relatedness), their mental health is likely to diminish. If a worker berates themselves for lacking competence in the workplace, evaluate their lack of autonomy in extreme ways, and views their lack of belonging as intolerable, then it is no wonder that they will feel a greater intensity of negative affect. Practically speaking, these results highlight a need to weaken irrational beliefs concerning psychological need satisfaction through rational emotive behaviour therapy (e.g., 19) and motivation based therapeutic techniques (8). Workforces can apply REBT to help workers weaken their irrational BPN beliefs in order to fortify them against instances when BPN’s are not satisfied (e.g., 87). Workers can be helped to understand that just because they *want* to be competent, this does not mean that they *must* be. This shift in thinking means that when basic needs are not satisfied (which is a certainty in an unpredictable work environment), they can respond more adaptively with less negative affect. Future research needs to further understand the influence of irrational beliefs on a multitude of mental and physical health variables in longitudinal, and experimental designs. But there seems to be little downside in helping workers to think more rationally.

There is a mass of empirical support for the positive influence of basic psychological need satisfaction on a multitude of variables (8), yet what is not clear is the influence of irrational beliefs that are attached to these needs (i.e., I must feel competent at work, and I am worthless as a person if I am not) on mental health. Based on the results of the present research, it seems to be the case that when one places irrational demands on satisfying basic psychological needs, and sees a lack of need satisfaction as terrible, this may propagate anxiety, depression, and negative emotions in the working individual. Expanding REBT and SDT theory, it seems as though irrational beliefs concerning need satisfaction can associate with negative mental health outcomes of employees as well as adolescents (18). As such, with this new ability to identify irrational beliefs concerning psychological needs in workers (using the RESD-W), adaptations to REBT practice can be made, incorporating strategies that both target psychological needs satisfaction as well as irrational beliefs concerning these needs. In other words, we can help workers to loosen their grip on irrational beliefs concerning their needs satisfaction, but also create environments that support the attainment of these needs (see 88, 89 for examples).

## Limitations

Much like all questionnaire-based research, the quality of the data is based on the honesty of respondents. Stigma associated with any mental health concerns in the workplace may have led to underreporting of mental ill-health (90). As such, organisations should work to reduce said stigma in order to reduce the prevalence of underreported ill-health (91). Honesty and authenticity is also key in the assessment of face validity – in that the respondents must be authentic in whether they perceived the item to be measuring what it intends to. Regarding validity, whilst the current paper did indicate some convergent and discriminant validity through interpreting correlations between RESD-W subscales and basic psychological needs support and positive work emotion, and through AVEs and SVs, future research needs to more clearly examine convergent and discriminant validity. Despite some positive indications, in truth there is currently limited evidence for the convergent and discriminative validity of the RESD-W. Furthermore, the concurrent validity evidenced in the present paper is only partially due to its non-temporal nature. In future research, the ability for RESD-W scores to predict changes in psychological distress outcomes would offer strongly evidence of concurrent validity.

We also used non-random sampling in our participant recruitment, which of course could lead to biases in our results. There is scope to further assess the validity of the RESD-W in this light and future research could apply random sampling and assess convergent and discriminant validity. Also, there was a significant subsample that were unemployed within this study. Whilst this can be interpreted as counterintuitive, we were interested in their beliefs about work, be it whether they were currently employed or not. Their beliefs regarding autonomy, competence and relatedness to work are still valid, as it is plausible that part of the reason that these respondents may be unemployed is due to issues related to irrational beliefs regarding autonomy, competence, and relatedness. Future research could compare RESD-W scores between employed and unemployed persons to understand potential differences.

There are also various ways in which researchers can approach statistical analysis procedures when undertaking psychometric validation. In the current paper we first employed EFA and then CFA using an independent sample as guided by Cabrera-Nguyen (35) and as conducted in recent similar research (52). But we could have employed confirmatory and exploratory structural equation modeling (ESEM) solutions. Indeed, ESEM is suited to validation studies when factors are expected to correlate (as evidenced in the present study), as it allows a thorough examination of individual items by freely estimating all cross-loadings of indicators (i.e., items) on all latent factors in measurement models (92; see also 93). Future research could take a different statistical approach to data collected for the RESD-W to examine alternative models and factor loadings, and could employ network analysis to better understand the underlying structure of the RESD-W and its associations with particular outcomes (94). Lastly, in Study 4 we used a BPN psychometric that was contextualised to the workplace due to the origin of data collection. Because this was contextualised to the workplace, and not initially validated in the workplace (i.e., with family and friendship groups), results concerning these markers should be interpreted with caution.

## Conclusions

This paper provides an initial development and validation of the RESD-W, in assessing irrational beliefs concerning the basic psychological needs of autonomy, competence and relatedness. The results identified a three-factor structure for the 16-item RESD-W. Analyses confirmed theoretical expectations and yielded good psychometric properties, but further research is needed. Based on the results, there is scope to further assess the validity of the measure, identify the effect of REBT on both irrational beliefs and basic psychological needs, and identify whether and to what extent irrational beliefs about competence, autonomy and relatedness influence mental and physical health variables. To this end, future studies could assess the validity and reliability of the RESD-W in different working samples across a wide array of occupational settings, and undertake longitudinal measurement of mental health and work productivity markers to elucidate the extent to which scores on the RESD-W predict changes in these important outcomes temporally.

## Data Availability

The raw data supporting the conclusions of this article will be made available by the authors, without undue reservation.
